# Histone Methyltransferase DOT1L Drives Recovery of Gene Expression after a Genotoxic Attack

**DOI:** 10.1371/journal.pgen.1003611

**Published:** 2013-07-04

**Authors:** Valentyn Oksenych, Alexander Zhovmer, Salim Ziani, Pierre-Olivier Mari, Jitka Eberova, Tiziana Nardo, Miria Stefanini, Giuseppina Giglia-Mari, Jean-Marc Egly, Frédéric Coin

**Affiliations:** 1 IGBMC, Department of Functional Genomics and Cancer, CNRS/INSERM/Université de Strasbourg, C. U. Strasbourg, France; 2 Université de Toulouse, UPS, IPBS, Toulouse, France; 3 Istituto di Genetica Molecolare, Consiglio Nazionale delle Ricerche, Pavia, Italy; Cologne Excellence Cluster for Cellular Stress Responses in Aging Associated Diseases, Germany

## Abstract

UV-induced DNA damage causes repression of RNA synthesis. Following the removal of DNA lesions, transcription recovery operates through a process that is not understood yet. Here we show that knocking-out of the histone methyltransferase DOT1L in mouse embryonic fibroblasts (MEF^DOT1L^) leads to a UV hypersensitivity coupled to a deficient recovery of transcription initiation after UV irradiation. However, DOT1L is not implicated in the removal of the UV-induced DNA damage by the nucleotide excision repair pathway. Using FRAP and ChIP experiments we established that DOT1L promotes the formation of the pre-initiation complex on the promoters of UV-repressed genes and the appearance of transcriptionally active chromatin marks. Treatment with Trichostatin A, relaxing chromatin, recovers both transcription initiation and UV-survival. Our data suggest that DOT1L secures an open chromatin structure in order to reactivate RNA Pol II transcription initiation after a genotoxic attack.

## Author Summary

Through the deformation of the genomic DNA structure, UV-induced DNA lesions have repressive effect on various nuclear processes including replication and transcription. As a matter of fact, the removal of these lesions is a priority for the cell and takes place at the expense of fundamental cellular processes that are paused to circumvent the risks of mutations that may lead to cancer. The molecular mechanism underlying transcription inhibition and recovery is not clearly understood and appears more complicated than anticipated. Here we analyzed the process of transcription recovery after UV-irradiation and found that it depends on DOT1L, a histone methyltransferase that promotes the reformation of the transcription machinery at the promoters of UV-repressed genes. Our discovery shows that transcription recovery after a genotoxic attack is an active process under the control of chromatin remodelling enzymes.

## Introduction

Short-wave UV light is a significant source of mutagenic and cytotoxic DNA damage. UV irradiation induces two major types of DNA lesions; the cis-syn cyclobutane-pyrimidine dimers (CPD) and the pyrimidine (6-4) pyrimidone photoproducts (6-4PP) [1]. Through the deformation of the DNA structure, these lesions have repressive effect on various nuclear processes including replication and transcription. As a matter of fact, the removal of these lesions is a priority for the cell and takes place at the expense of fundamental cellular processes that are paused to circumvent the risks of mutations that may lead to cancer. The molecular mechanism underlying transcription inhibition and recovery is not understood yet but it includes proteins such as CSB, a member of the SWI2/SNF2 family of chromatin remodeling proteins, which promote transcription re-initiation at the promoters of UV-repressed genes [2], [3].

UV lesions are removed from DNA by the nucleotide excision repair (NER) mechanism through two sub-pathways. The general global genome repair (GG-NER) removes DNA damage from the entire genome, while the transcription-coupled repair (TC-NER) corrects lesions located on actively transcribed genes [4]. In TC-NER, an elongating RNA polymerase II (RNA Pol II) stalled by a lesion triggers efficient repair of the cytotoxic damage that blocks transcription, while lesion elsewhere in the genome are detected by the XPC/hHR23B complex for GG-NER [5]. Then, both sub-pathways funnel into a common process involving XPA, RPA, TFIIH, XPG and XPF-ERCC1 to excise damaged oligonucleotides from DNA.

Post-translational histone modifications modulate promoter activity. Histone acetylation, phosphorylation, ubiquitination, and methylation dictate the transcriptional fate of any given locus [6]. Inactive heterochromatin is associated with high levels of methylation at H3K9, H3K27, and H4K20 residues and low levels of acetylation, while actively transcribed euchromatin shows a high level of acetylation of H4K16 and H4K20 and methylation of H3K4, H3K36, and H3K79 residues [7], [8].

The *dot1* gene (disruptor of telomeric silencing-1), also called *kmt4* (lysine methyltransferase-4), encodes a protein that exclusively methylates lysine 79 of histone H3 (H3K79) [9], [10], [11]. Unlike most modified histone residues that are located within the N-terminal tail, H3K79 is found within the globular core of the histone octamer [12]. The Dot1 protein is the only histone lysine methyltransferase that does not contain the conserved SET domain but exhibits a methyltransferase fold that is responsible for its activity [13], [14]. In mammals, several studies have shown that DOT1L (the Dot1 homolog) exists in a complex that trimethylates H3K79 and that contains several myeloid/lymphoid or mix-lineage leukemia fusion partners, such as MLLT1, 2, 3 or 10 [15]. More recently, DOT1L was shown to be involved in cell cycle progression, the control of the differentiation of pluripotent cells [16] and leukemogenesis [17].

In addition to these roles in fundamental cellular processes, several lines of evidence suggest that DOT1L plays an important role in genomic stability. DOT1L has been reported to favor the recruitment of double strand breaks sensor 53BP1 to DNA lesions [18]. Dot1 is known to be required for the activation of the *RAD9/RAD53* checkpoint function by UV and γ-radiation [19], [20], [21]. Dot1 also plays a role in the yeast cellular response to UV damage but its specific function in this mechanism is unclear [22].

Here, we provide evidence that the mammalian DOT1L protein is required to re-initiate transcription after UV irradiation. Knocking-out of DOT1L results in high sensitivity to UV irradiation in mouse embryonic fibroblasts (MEF), but preserves an accurate repair of (6-4)PP lesions. Instead, MEF^DOT1L^ are unable to recover transcription of constitutively expressed genes after UV irradiation. Using fluorescence recovery after photobleaching (FRAP), we have shown that DOT1L regulates the recruitment of RNA Pol II to chromatin after UV irradiation. Applying chromatin immunoprecipitation assay, we additionally revealed that DOT1L triggers the formation of the pre-initiation complex (PIC) to the promoters of UV-repressed genes, as well as the appearance of active transcriptional marks on histones. Altogether, our results highlight a new role for DOT1L in the maintenance of an open chromatin structure in order to reactivate the formation of the transcription machinery on the promoter of constitutive genes after a genotoxic attack.

## Results

### DOT1L deficient mammalian cells are UV-sensitive

**Figure 1:**
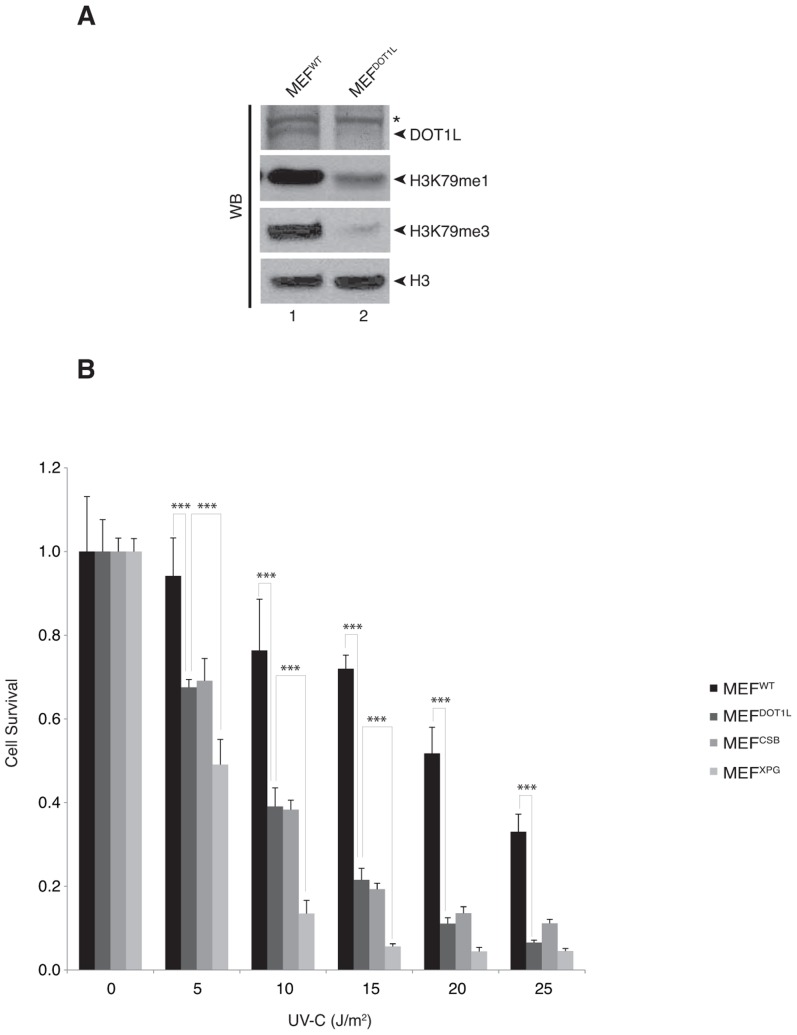
DOT1L is involved in UV-survival in mammalian cells. (**A**) Fifty µg of whole cell extracts from MEF^WT^ or MEF^DOT1L^ cells were resolved by SDS-PAGE and Western-blotted for DOT1L. Asterisk indicates an unspecific cross-reacting band. Ten µg of fractions from histone acid-extraction performed on MEF^WT^ or MEF^DOT1L^ cells were resolved by SDS-PAGE and Western-blotted for H3, H3K79me1 or H3K79me3. (**B**) MEF^WT^, MEF^DOT1L^, MEF^XPG^ and MEF^CSB^ cells were tested in a UV-survival assay. Cells were treated with increasing dose of UV-C and cell survival was determined 96 hours later, as detailed in the Experimental Procedures. Data were normalized to the mock treatment controls (as value of 1). The values are the means of three independent experiments (± SD). P-value was extrapolated for MEF^DOT1L^/MEF^WT^ or MEF^DOT1L^/MEF^XPG^ and indicated on the graph (***<0.005).

To investigate the role of DOT1L in the repair of UV-induced DNA damage, we used knocked-down MEF^DOT1L^ cells carrying a homozygous gene trap insertion in *Dot1l* [23]. Together with an absence of *Dot1l* protein expression, the mono- and tri-methylation of H3K79 were strongly reduced in MEF^DOT1L^ (Figure 1A). In a UV-C survival assay, MEF^DOT1L^ cells were more sensitive to irradiation as compared to control MEF^WT^ but less than MEF^XPG^, knocked-out for the NER factor XPG and deficient both for GG- and TC-NER [24] (Figure 1B). However, MEF^DOT1L^ showed similar UV-C sensitivity than the MEF^CSB^ cells, knocked-out for the CSB protein involved only in TC-NER. Note that tri-methylation of H3K79 was similar in MEF^WT^, MEF^XPG^ and MEF^CSB^ (Supplemental Figure S1A). Knocking-down of DOT1L expression in HeLa cells using siRNA (Supplemental Figure S1B) recapitulated the mild UV-sensitivity of the MEF cells compared to the high UV-sensitivity induced by the knocked-down of the NER factor XPA (Supplemental Figures S1B–C). These data indicate that DOT1L deficiency induces UV-sensitivity in mammalian cells.

**Figure 2:**
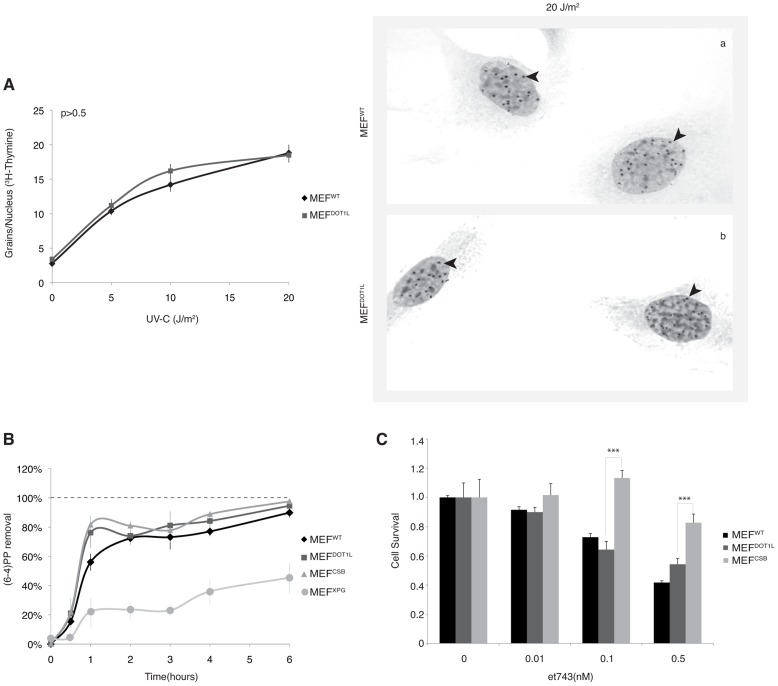
DNA repair takes place in the absence of DOT1L. (**A**) Unscheduled DNA Synthesis (UDS) assay. Left panel; UDS expressed as mean number of autoradiographic grains/nucleus. UDS was measured by incubating MEF^WT^ or MEF^DOT1L^ with radioactive [^3^H]thymidine before treatment with increasing doses of UV-C light and autoradiography [25]. The values are the means of two independent experiments +/−SEM. AUC for each curve were determined and p-value was extrapolated using a t-test. Right Panel; Autoradiogram of the UDS experiment performed at 20 J/m^2^ in either MEF^WT^ (a) or MEF^DOT1L^ (b) cells. Arrows indicate radioactive dots. (**B**) (6-4)PP removal was carried out in MEF^WT^, MEF^DOT1L^, MEF^XPG^ and MEF^CSB^, harvested at different time points after UV irradiation at 20 J/m^2^. Cells were labeled with an anti-(6-4)PP antibody and signals were measured using IN Cell 1000 analyzer (GE Healthcare). Graph represents the % of lesions removed from the genome at different time points. The values are the means of three independent experiments (± SD). For each time point, a mean of 4000 cells has been analysed. (**C**) MEF^WT^, MEF^DOT1L^ and MEF^CSB^ cells were treated with increasing concentration of et743 and cell survival was determined 96 hours later, as detailed in the Experimental Procedures. Data were normalized to the mock treatment controls (as value of 1). The values are the means of three independent experiments (± SD). P-value is indicated (***<0.005) and was determined between MEF^DOT1L^ and either MEF^CSB^.

We further investigated whether DOT1L affected UV-C survival by sustaining the repair of UV-induced DNA damage. We performed unscheduled DNA synthesis assay (UDS), which is mainly a measure of the GG-NER efficiency [25]. The UDS of MEF^DOT1L^ was identical to that of the MEF^WT^ (Figure 2A). We also used an assay based on immunofluorescence coupled to quantification of DNA lesions directly in cell nucleus to measure the removal of the (6-4)PPs, the main UV-induced DNA lesions (see experimental procedures). MEF^XPG^ cells showed low removal of (6-4)PP along the time course of repair, compared to MEF^WT^ or MEF^CSB^ in which lesions were rapidly removed (Figures 2B). When measured in MEF^DOT1L^ cells, the removal rate of (6-4)PP lesions was identical to that of MEF^WT^ (Figures 2B), which implied that the absence of DOT1L does not impair GG-NER.

**Figure 3:**
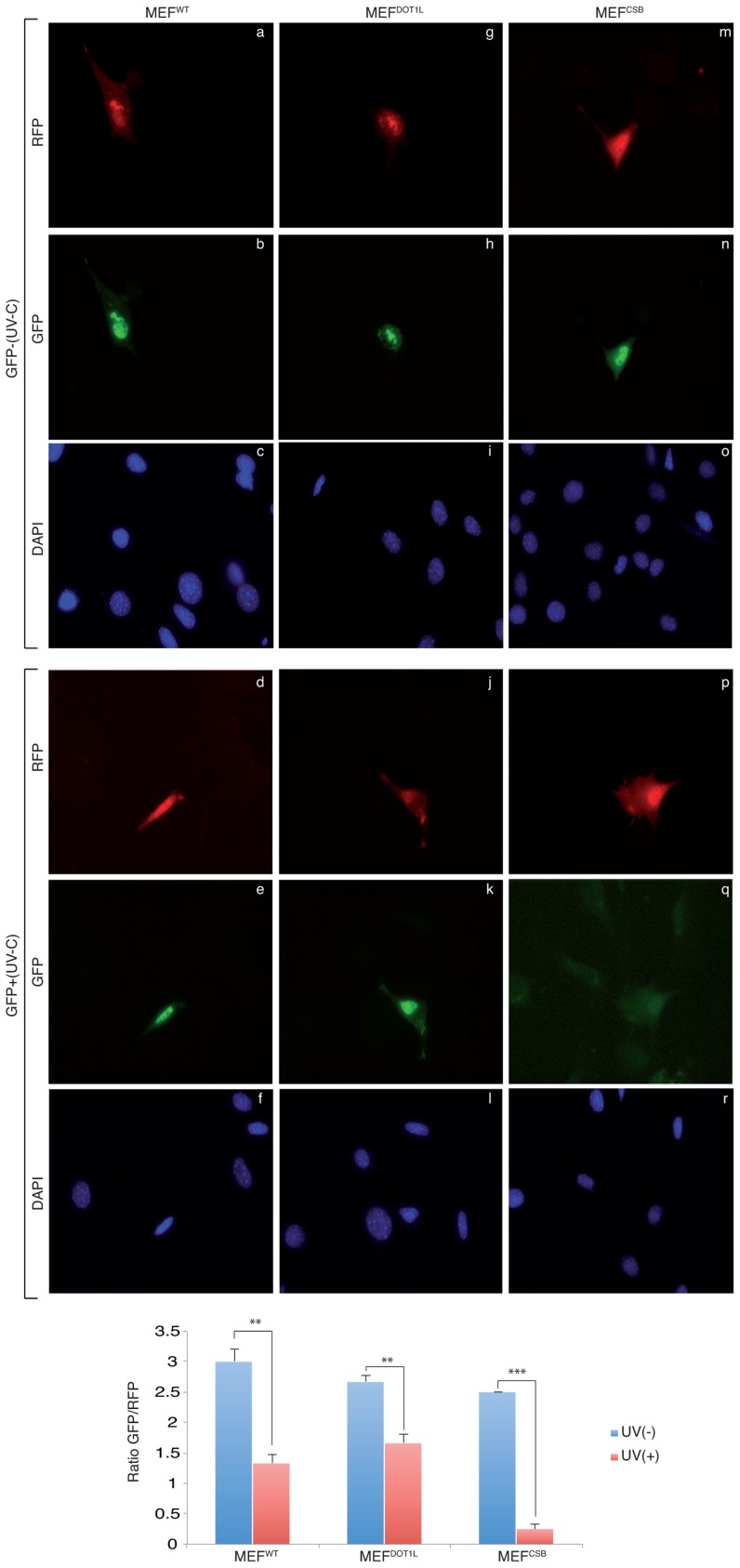
Host cell reactivation assay in MEF cells. MEF^WT^, MEF^DOT1L^ and MEF^CSB^ were transfected with mock-treated (panels a–c, g–i, m–o) or irradiated (UV-C, 600 J/m^2^) (panels d–f, j–l, p–r) pEGFP reporter plasmid in combination with a non-irradiated pRFP reporter plasmid used as control. Twelve hours post transfection, GFP and RFP expression were determined. A ratio of 3/1 (pEGFP/pRFP) was used during transfection to ensure that any cell expressing the RFP expresses also the GFP. P-value was extrapolated using t-test (**<0.05, ***<0.005).

To determine whether MEF^DOT1L^ were able to perform TC-NER, we performed two sets of experiments. First we measured cell survival following incubation with Ecteinascidin 743 (et743), an anti-tumor drug that shows cytotoxicity effect only on human TC-NER proficient cells [26]. In our experimental conditions, knocking-down of the TC-NER factor CSB in MEF cells resulted in et743 resistance, compared to MEF^WT^ cells (Figure 2C). In contrast, MEF^DOT1L^ cells showed sensitivity to et743 to a level equivalent to that of MEF^WT^ cells (Figure 2C). Next, we performed a host cell reactivation assay [27]. We employed a dual GFP/RFP readout with a UV-damaged plasmid (600 J/m^2^, 3 Kb) containing a GFP-tagged reporter and an undamaged plasmid containing an RFP-tagged reporter transiently transfected into MEF cells. Recovery of GFP-reporter expression, 12 hours post-transfection, was efficient in both MEF^WT^ (Figure 3, panels a–f) and MEF^DOT1L^ (Figure 3, panels g–l) cells but not in the TC-NER deficient MEF^CSB^ cells (Figure 3, m–r). Overall, these results suggest that the absence of DOT1L induces sensitivity to UV irradiation that is not the consequence of a defect in GG- or TC-NER.

### DOT1L promotes transcription recovery after UV irradiation

**Figure 4:**
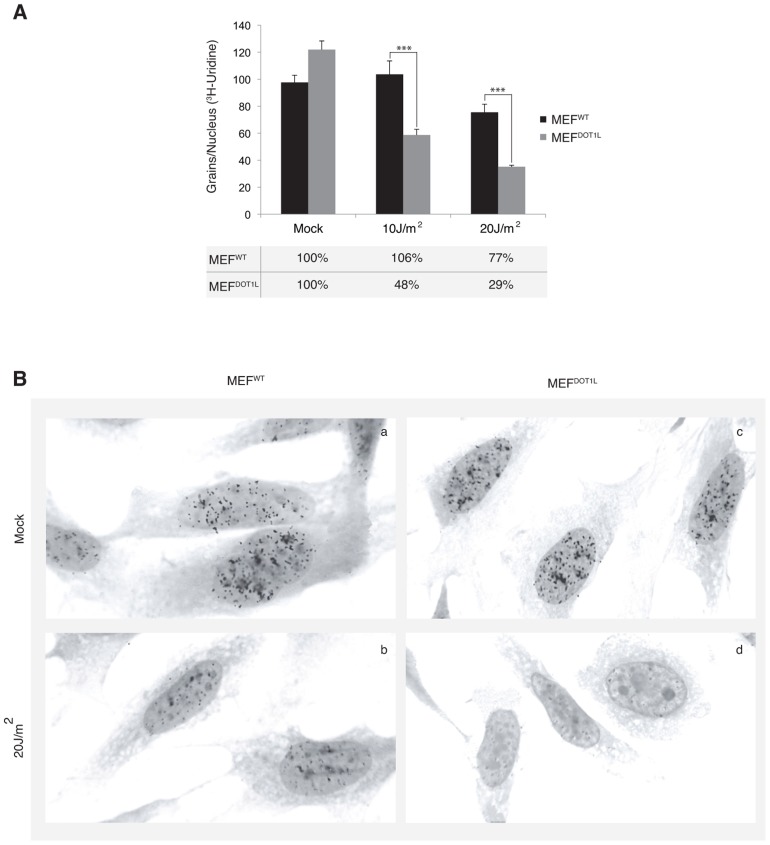
Deficient transcription recovery after UV irradiation in the absence of DOT1L. (**A**) Recovery of RNA synthesis assay (RRS). The mean numbers of auto-radiographic grains per nucleus of mock treated or UV-irradiated cells (10 or 20 J/m^2^) from two independent experiments are expressed (± SEM, measured on at least 150 cells). Under the graph, the results are expressed as percentage of grains per nucleus relative to mock treated cells. (**B**) Autoradiogram of an RRS experiment. Twenty four hours after UV irradiation (20 J/m^2^), MEF^WT^ (panels a–b) or MEF^DOT1L^ (panels c–d) cells were pulse labeled 30 minutes with [^3^H]uridine followed by autoradiography. P-value was extrapolated using t-test (**<0.05, ***<0.005).

Next we aimed to determine the global RNA synthesis of MEF cells after irradiation using the recovery of RNA synthesis (RRS) assay [25]. Cells were UV-irradiated with 10 or 20 J/m^2^ and incubated with radioactive [^3^H]uridine during a 30 minutes pulse performed before and 24 hours after irradiation. Mock-treated MEF^WT^ and MEF^DOT1L^ cells showed similar levels of RNA synthesis, visualized by equivalent number of black dots in their nuclei (around 100 dots/nucleus, Figure 4A and 4B, compare panels a and c). In contrast, we observed a marked deficiency in RNA synthesis in MEF^DOT1L^ cells, 24 hours after UV-C treatment, as compared to MEF^WT^ (Figure 4A and 4B, compare panels b and d). We estimated the residual transcription activity in the MEF^DOT1L^ cells to 30% of that of the mock-treated cells, 24 hours after irradiation with 20 J/m^2^.

### DOT1L ensures RNA Pol II binding to chromatin after UV irradiation

**Figure 5:**
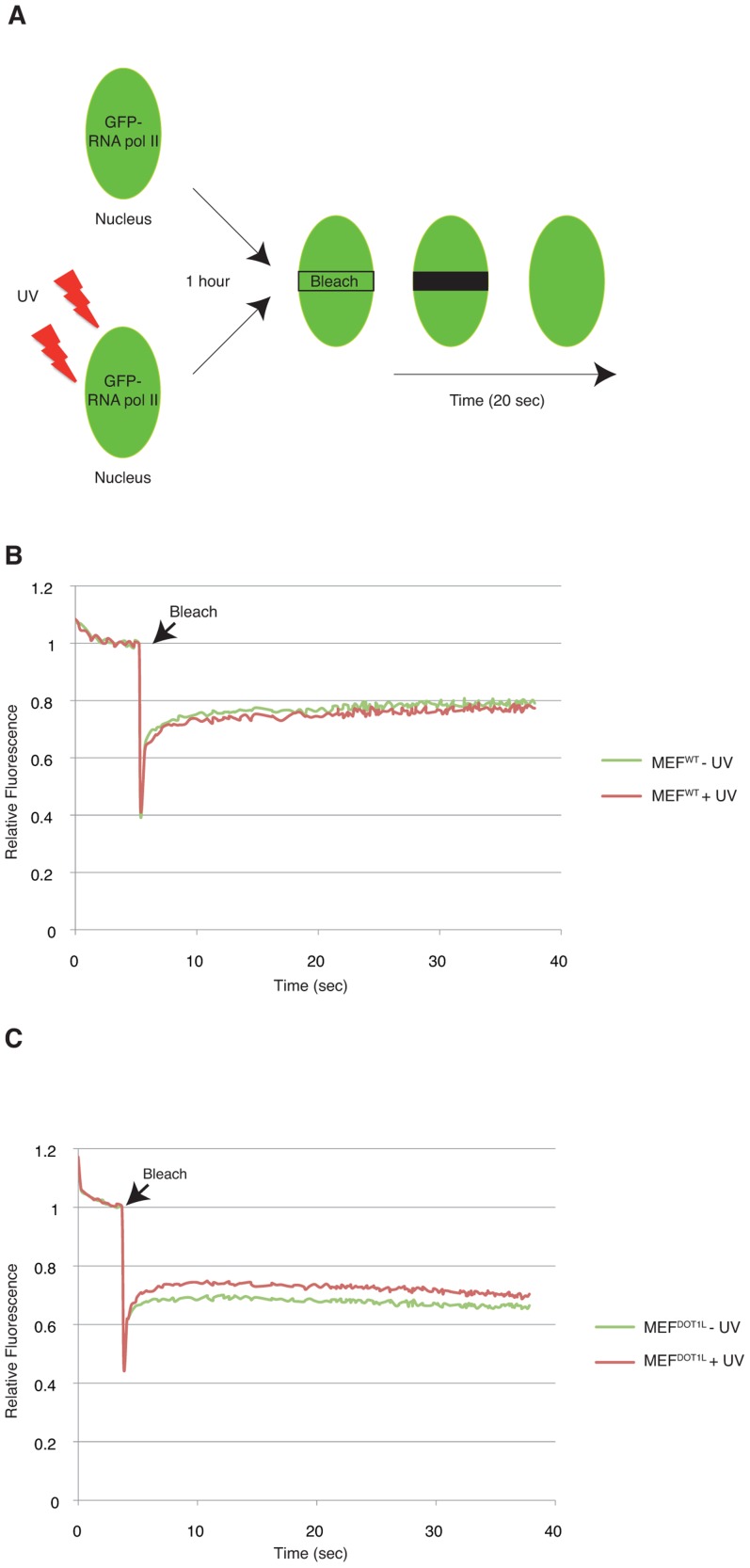
DOT1L ensures binding of RNA Pol II to chromatin after UV irradiation. (**A**) Schematic diagram showing the FRAP assay. Cells were transfected with a GFP-RNA Pol II construct (on RPB1). A small region in the middle of the nucleus was bleached and the subsequent fluorescence recovery was followed in time. When indicated, cells were UV-irradiated, 1 hour before the photobleaching. (**B–C**) FRAP curves of RNA Pol II-GFP protein stably expressed in either MEF^WT^ (**B**) or MEF^DOT1L^ (**C**) cells untreated (green) or treated (red) with UV (16 J/m^2^), 1 hour before photobleaching. Cells were photobleached with a 488 nm laser at maximum power 4 sec after the beginning of the acquisition. One image per 20 msec was taken during 20 sec in the photobleached area. SEM is within the range of 1% and is shown is supplemental Data 1.

To unveil the molecular mechanisms that led to the inhibition of transcription in MEF^DOT1L^ cells after UV irradiation, we examined live-cell protein mobility of RNA Pol II by fluorescence recovery after photobleaching (FRAP). In brief, a small region in the middle of the nucleus was bleached and the subsequent fluorescence recovery was measured in time (Figure 5A). For that purpose, the largest RNA Pol II subunit RPB1 was fused with GFP and expressed either in MEF^WT^ or MEF^DOT1L^. In these conditions, we found an equivalent mobility of RNA Pol II in mock-treated and UV-irradiated (UV-C, 16 J/m^2^) MEF^WT^ (Figure 5B and Supplemental Data S1) (T-test = 3.4E-2). In marked contrast, FRAP analysis of UV-damaged MEF^DOT1L^ cells revealed a significant increase in fluorescence recovery when compared to mock-treated cells (Figure 5C and Supplemental Data S1) (T-test = 1.4E-5), indicating that a fraction of RNA Pol II became mobile in the absence of DOT1L, after UV irradiation.

### Defect in transcription initiation after UV irradiation in cells depleted of DOT1L

**Figure 6:**
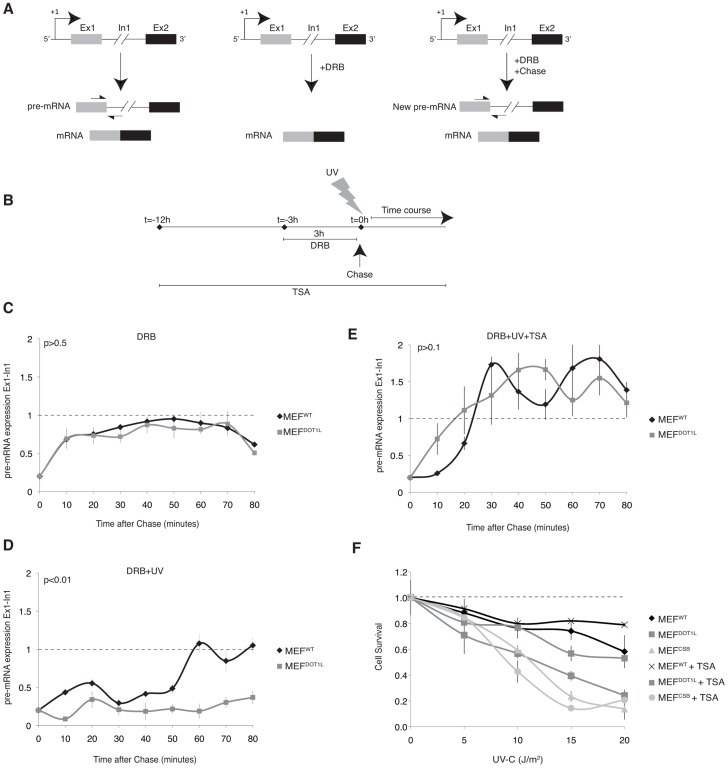
Inhibition of the initiation-to-elongation transition phase in the absence of DOT1L, after UV irradiation. (**A**) Schematic representation of measuring the initiation-to-elongation transition rate of RNA Pol II transcription *in vivo* on endogenous genes. We reversibly blocked gene transcription by incubating cells with DRB. Cells are depleted of their pre-mRNA pool within few hours of incubation with the drug [28]. Following the chase of DRB, RNA Pol II is released from promoter-proximal regions and newly synthesized pre-mRNA appear; the level of pre-mRNA is measured using oligonucleotides targeting respectively the exon/intron junctions of the gene. (**B**) Schematic diagram showing the experimental approach used to measure the rate of initiation-to-elongation transition phase by RNA Pol II after UV irradiation. MEF cells were treated with DRB for 3 hours before chase and addition of fresh medium at t = 0 hour. When indicated, cells were UV irradiated at t = 0 hour, before the addition of fresh medium or treated with TSA for 12 hours before the addition of DRB. (**C**) Expression levels of the newly synthesized Exon1 of the *Utrophin* gene in MEF^WT^ and MEF^DOT1L^ cells treated with 100 µM of DRB for 3 hours before the addition of fresh medium. The cells were harvest at intervals of 10 minutes for RNA isolation and qRT-PCR was performed using oligonucleotides targeting respectively the Exon1 and Intron1 of the gene. Relative expression values compared to mock treated cells are plotted against time (±SD). AUC was determined and p-value was extrapolated using t-test. (**D**) Expression levels of the newly synthesized Exon1 of the *Utrophin* gene in either MEF^WT^ or MEF^DOT1L^ irradiated with UV-C (15 J/m^2^) after treatment as in (**C**). AUC was determined and p-value was extrapolated using t-test. (**E**) Expression levels of the newly synthesized Exon1 of the *Utrophin* gene in either MEF^WT^ or MEF^DOT1L^ treated with TSA (20 nM) for 12 hours before addition of DRB for 3 hours and UV-C irradiation (15 J/m^2^). TSA was maintained in the medium during DRB treatment and time course. AUC was determined and p-value was extrapolated using t-test. (**F**) MEF^WT^, MEF^CSB^ and MEF^DOT1L^ were irradiated with increasing doses of UV-C light. Cell survival was determined 96 hours later, as detailed in the Experimental Procedures. Data were normalized to the mock treated controls (as value of 1). Means of three independent experiments are shown (± SD). When indicated, cells were treated with TSA (10 nM), 12 hours before UV irradiation, and TSA was maintained for the time of the experiment.

We next wondered whether DOT1L was required to mobilize RNA pol II either during the initiation or elongation steps of transcription. For this purpose, we examined the step of transition from initiation to elongation by the RNA Pol II *in vivo* on an endogenous mouse gene. Briefly, we reversibly blocked gene transcription by incubating cells with the P-TEFb inhibitor DRB (5,6-dichloro-1-β-D-ribobenzimidazole), which inhibited the transition from initiation to elongation but did not block elongation of ongoing mRNA transcripts [28] (Figure 6A). Following the removal of DRB, RNA Pol II was released from promoter-proximal regions and the level of newly synthesized pre-mRNA had been measured owing to the presence of exons and introns. We measured the transition from initiation to elongation on the *Utrophin* gene that possesses a very short Exon1 (170 bp). We estimated that an average of one transcription blocking lesion (CPD or (6-4)PP) was created per 5 kb of DNA at 20 J/m^2^ [29], indicating that less than 5% of cells harbor a UV-lesion in this exon following a dose of 15 J/m^2^ of UV-C. Therefore, any significant inhibition of transcription initiation cannot be explained by the blockage of RNA Pol II in front of a lesion in Exon 1.

We treated MEF cells for 3 hours with DRB and extracted RNA at 10 minutes intervals after the removal of the drug (Figure 6B). Next, we performed RT-PCR using primers spanning Exon1-Intron1 junctions to detect newly synthesized pre-mRNA of *Utrophin* gene. In the absence of genotoxic stress, MEF^WT^ and MEF^DOT1L^ were both able to recover transcription of the Exon1 region within 10 to 20 minutes after DRB removal (Figure 6C), indicating that the transcriptional initiation rate in MEF^WT^ and MEF^DOT1L^ was identical, in absence of a genotoxic attack. Then, we irradiated MEF cells with UV-C (15 J/m^2^) after the DRB treatment (Figure 6B). In these experimental conditions, MEF^WT^ were able to recover transcription of the Exon1 within 60 minutes after removal of DRB and UV-treatment, while MEF^DOT1L^ showed no recovery of Exon1 transcription even after 80 minutes (Figure 6D).

Since DOT1L was shown to be involved in chromatin remodeling, we next tested whether chromatin relaxation could overcome the absence of DOT1L. For that purpose, we treated MEF^DOT1L^ with Trichostatin A (TSA, 20 nM), a class I histone deacetylase (HDAC) inhibitor that relaxed chromatin (Figure 6B). Following TSA treatment, we observed a recovery of Exon1 pre-mRNA expression in MEF^DOT1L^, which peaked between 30 to 40 minutes after UV irradiation (Figure 6E) and paralleled the transcription of Exon1 in MEF^WT^ cells. Together with this recovery, pre-treatment of MEF^DOT1L^ with TSA (10 nM) induced a potent recovery of UV survival (Figure 6F) suggesting that transcription inhibition in MEF^DOT1L^ was indeed responsible for the UV-sensitivity of these cells. Pre-treatment of the TC-NER deficient MEF^CSB^ with TSA did not modify their UV-sensitivity (Figure 6F). Altogether, these data suggested that DOT1L allowed RNA Pol II transcription re-initiation after UV irradiation.

### Repressive chromatin marks in cells depleted of DOT1L after UV irradiation

**Figure 7:**
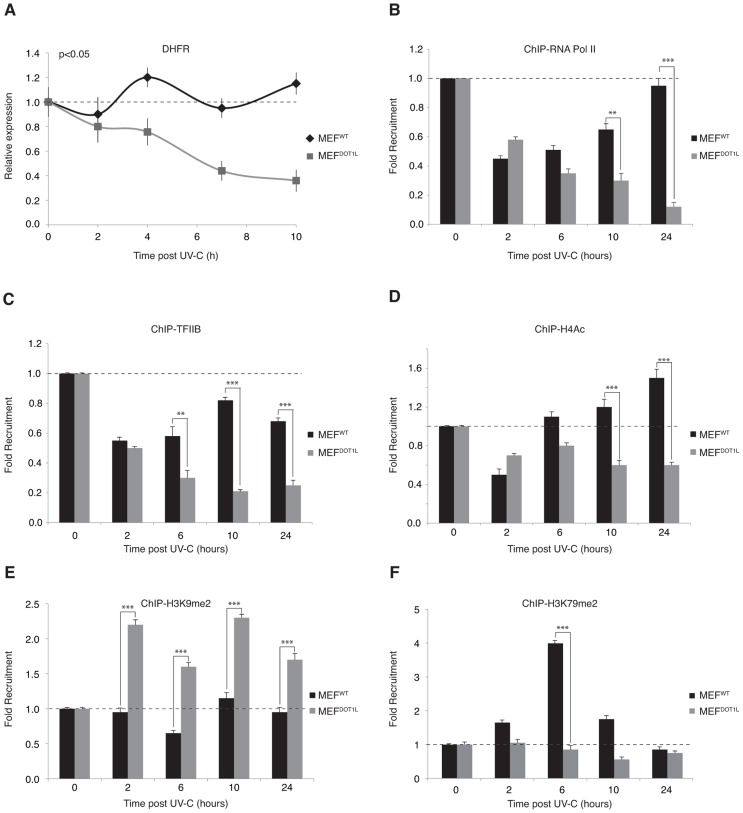
Pre-initiation complex assembly and chromatin modification after UV irradiation. (**A**) Determination of the relative mRNA expression level of *DHFR* gene in MEF^WT^ and MEF^DOT1L^ after UV irradiation (10 J/m^2^) performed after reverse transcription with random hexameres. The values are the means of three independent experiments (± SD). AUC was determined and p-value was extrapolated using t-test. (**B–F**) Time-dependent occupancy of RNA Pol II (**B**), TFIIB (**C**), H4Ac (**D**), H3K9me2 (**E**) and H3K79me2 (**F**) at the promoter of the *DHFR* gene following UV irradiation (10 J/m^2^). Soluble chromatin was prepared from MEF^WT^ and MEF^DOT1L^ cells at indicated time points after UV-C treatment and subjected to ChIP assay using the indicated antibodies. Real-time PCR using specific primers was performed to test the relative enrichment at the proximal promoter of the DHFR gene. The results are expressed as folds of enrichment relative to the untreated cells. The values are the means of a triplicate experiment (± SD). P-value was extrapolated using t-test (**<0.05, ***<0.005).

The above data prompted us to perform a detailed analysis of the PIC formation on the promoter of UV-repressed genes throughout the time, after irradiation. We studied the promoter of the several housekeeping genes such as *DHFR* (dihydrofolate reductase), *B2M* (beta-2-microglobulin) *or KLF7* (Kruppel-like factor 7) that we used as models for assembly/disassembly of the PIC after UV-irradiation. Using chromatin immunoprecipitation (ChIP), we observed a slight decrease in both RNA Pol II (Figures 7B and Supplemental Figure S2) and basal transcription factors (Figure 7C) occupancy at these promoters in UV-irradiated MEFs, 2 hours post-UV irradiation. The transcription machinery started to re-assemble on the promoter between 6 and 10 hours after UV irradiation and the steady state level of mRNA did not vary significantly with time in the wild-type situation (Figure 7A). In contrast, the basal transcription machinery did not re-form on the UV-repressed gene promoter throughout the entire time course in MEF^DOT1L^ cells (Figures 7B, 7C and Supplemental Figure S2). In line with these observations, the steady state level of mRNA decreased progressively after UV irradiation in MEF^DOT1L^ cells (Figure 7A). This observation is in agreement with results obtained in Figure 4 in which we measured global transcription.

We then analyzed chromatin modifications associated with active or inactive chromatin in the promoter region of the *DHFR* gene. We observed an acetylated histone H4 pattern that followed that of the RNA Pol II, with a first phase of decrease 2 hours post-UV, and a phase of increase from 6 to 24 hours (Figure 7D). In contrast, no phase of recovery was observed in MEF^DOT1L^ cells (Figure 7D). Di-methylation of H3K9 residue, a mark of transcription silencing, was stable in MEF^WT^ throughout the time course after UV-treatment, while it increased just after irradiation in MEF^DOT1L^ (Figure 7E). Finally, di-methylation of H3K79, performed by DOT1L, was observed transiently in the promoter region of *DHFR* and peaked 6 hours after UV irradiation (Figure 7F), when RNA Pol II and TFIIB started to come back to the *DHFR* promoter in MEF^WT^ cells. Altogether these data suggested that DOT1L was required to drive recovery of PIC formation at the promoters of UV-repressed genes.

## Discussion

Transcription inhibition and the subsequent recovery that operate after a genotoxic attack are thought to limit the risks that lesions represent for the genome. The molecular mechanisms that are responsible for the turn-off/turn-on of transcription after DNA damage are not well understood. Here we show that the methyltransferase DOT1L is required for the re-initiation of transcription by triggering re-formation of the transcription machinery at the promoter of UV-repressed genes. Our data supports the hypothesis that transcription recovery after a genotoxic attack is an active process involving specific actors insuring not only the repair of the DNA but also the recovery of fundamental cellular processes such as transcription.

### DOT1L participates in UV-survival but not in DNA repair

Our study demonstrated that disruption of DOT1L caused hypersensitivity to UV irradiation in mammalian cells. There are several potential mechanisms that could explain the increased sensitivity to UV irradiation conferred by DOT1L depletion. If it was directly involved in DNA repair, its absence may results in increased levels of DNA damage, leading to cellular apoptosis. A function for yeast DOT1 in GG-NER has been described recently [30]. However, we did not find any DNA repair defect associated with the absence of DOT1L in mammalian cells. Indeed, MEF cells depleted of DOT1L exhibited normal UDS level, an assay that mainly measured GG-NER. Furthermore, these cells repaired (6-4)PP, the best UV-induced NER substrate, at the same rate than wild-type cells. To show that MEF^DOT1L^ cells were also proficient in TC-NER, we used two strategies. First we made use of the sensitivity of cells to the drug et743, a natural marine product isolated from the Caribbean see squirt. Antiproliferative activity of et743 was shown to be dependent on active TC-NER [26]. In our experimental conditions, we observed that MEFs knockdown for CSB, one of the two TC-NER specific factors, exhibited a higher resistance to et743 treatment than MEF^WT^, confirming previous observation obtained with patient cells [26]. Using et743 on MEF^DOT1L^ cells, we observed that these cells were as sensitive to treatment as MEF^WT^. In a second set of experiments, we measured the capacity of MEF^DOT1L^ to drive the recovery of a reporter expression construct previously exposed to UV-irradiation. In mouse-derived cells, the reactivation depends both on efficient GG- and TC-NER activities as illustrated by the absence of transcription recovery in MEF^CSB^ cells observed both in our study and in other reports [31]. However, MEF^DOT1L^ showed a full recovery of reporter expression. Finally, the treatment of MEF^DOT1L^ with TSA induced a recovery of UV-survival while treatment of MEF^CSB^ did not. We concluded from these cellular observations summarized in Table 1 that it was unlikely that UV-irradiation sensitivity in DOT1L-deficient mammalian cells was due to a GG-NER or TC-NER defect.

**Table 1:** Cellular characteristics of MEF cells.

Cell lines	Cell survival to UV-C	RCS^1^ following TSA treatment	Cell survival to et743	UDS	(6-4)PP removal	RRS	HCRA^3^
MEF^WT^	+	+	−	+	+	+	+
MEF^DOT1L^	−	**+**	**−**	+	+	−	**+**
MEF^CSB^	−	**−**	**+**	+	+	−2	**−**
MEF^XPG^	–	ND	ND	ND	−4	ND	ND

1 - Recovery of Cell Survival.

2 - As observed in [38].

3 - Host Cell Reactivation Assay.

4 - As observed in [39].

The cellular characteristics of MEF cells studied in this report are summarized. In bold we highlighted the characteristics of MEF^DOT1L^ that differ from MEF^CSB^.

### DOT1L controls transcription recovery after UV irradiation

Alternatively, DOT1L may serve to reactivate global mRNA synthesis after UV irradiation. Transcriptional arrest has been shown to lead to a highly cytotoxic cellular response to stress [5]. This response has multiple causes and is likely not only the result of DNA lesions that block RNA Pol II in elongation. Previous studies have challenged the relationship between efficient repair of a lesion in the transcribed strand of active genes and the restoration of DNA damage inhibited transcription. For instance, cells carrying mutations in CSB were unable to recover lesion-inhibited transcription while they efficiently repaired acetylaminofluorene lesions in transcriptionally active genes [32]. In addition, CSB was shown to accumulate at the promoters of UV-repressed genes, where it stimulated the recovery of transcription independently of the presence of lesions [3]. This finding led to the hypothesis that removal of transcription blocking lesions was insufficient to restore transcription after DNA damage and that in addition, chromatin changes in the promoters of UV-repressed genes may be required. We performed RNA-sequencing on MEF^WT^ and MEF^DOT1L^ after UV-irradiation (unpublished Data). However, RNA-sequencing measures steady-state level of individual mRNA but does not provide information on the de novo RNA activity. To measured the global level of newly synthetized RNA we used the RRS assay and we observed a general inhibition of de novo RNA synthesis in cells depleted of DOT1L; 30% of residual transcription activity was detected 24 hours post irradiation (20 J/m^2^). This inhibition was confirmed at the single gene level since *DHFR* showed a similar level of inhibition.

### DOT1L allow PIC formation on UV-repressed gene promoters

Early steps of mRNA expression include formation of PIC, transcription initiation and escape of RNA Pol II from the promoter to the elongation step. Using an assay that measured the transcription of the newly synthetized first exon of *Utrophin* gene model *in vivo*, we demonstrated that the transition from initiation to elongation was deficient in the absence of DOT1L after UV. We further analysed both PIC occupancy and the chromatin modifications on the promoter of a UV-repressed gene after irradiation and have shown that this promoter was temporally depleted of basal transcription factors in the first hours after irradiation in wild-type cells. Six to ten hours after UV irradiation, when DNA repair took place, the PIC occupancy recovered completely in the wild-type situation. In the absence of DOT1L, RNA Pol II and TFIIB did not get back to the promoter of our UV-repressed gene model, and heterochromatin marks such as methylation of the H3K9 residue appeared. FRAP experiment using GFP-tagged RPB1 subunit of RNA Pol II showed that a fraction of RNA Pol II became mobile after UV-irradiation in the absence of DOT1L, indicating that dissociation of RNA Pol II from chromatin of UV-repressed genes after irradiation in the absence of DOT1L is a broad phenomenon.

Altogether, these data suggest that DOT1L favors an opened chromatin structure around the promoter of UV-repressed genes to allow transcription re-initiation. In line with this hypothesis, H3K79me2, the mark of DOT1L activity, was accumulating transiently on the promoter in wild-type cells after UV irradiation. In addition, the absence of DOT1L was circumvented by the class I HDAC inhibitor TSA that relaxes chromatin. TSA restored both transcription initiation and UV-survival of MEF^DOT1L^ cells, creating thus a link between these two events.

Among the sites of histone methylation, H3K79 is unique as it is not located within the H3 N-terminal tail domain but in the core region. Specifically, this methylation occurs on the surface of the nucleosome and may serve as a platform to recruit additional chromatin modifiers and DNA damage response factors [12]. On the other hand, regions of chromatin where transcription is repressed are depleted of H3K79 methylation, indicating that silencing of chromatin probably requires hypomethylation of H3K79 [11], [33]. The mechanism that links euchromatin to H3K79 methylation is not fully understood but it is believed that in addition to recruiting chromatin modifiers, this histone mark plays an important role in confining the Sir proteins to heterochromatic regions [11], [34]. In yeast, Sir3 binds to nucleosomes containing deacetylated histone H4K16 and promotes spreading of heterochromatin along the chromatin [35]. Based on these observations and our data, we propose that RNA Pol II re-accumulation at promoters after UV irradiation depends on the chromatin changes orchestrated by DOT1L, including the emergence of active chromatin transcription marks. In the absence of DOT1L, facultative heterochromatin marks such as H3K9me2 appear and RNA Pol II does not get back to the promoters. Through the recruitment of chromatin modifiers and subsequent histone modifications, DOT1L serves to limit the spreading of heterochromatin to UV-repressed genes immediately after irradiation and to allow re-association of the basal transcription machinery on the promoters of these genes to re-activate their transcription. Finally, one can wonder how specific is the requirement of DOT1L for the transcription recovery? We tested MYST2 and G9a, an H4 acetyl-transferase and an H3K4 methyltransferase respectively, but knocking down these chromatin remodelers did not induce any increase in UV sensitivity in Hela cells (Data not shown). Although this result shows that all transcriptional chromatin remodelers are not required to reactive transcription after DNA repair we believe that our observation will lead to the identification of additional factors relevant for the regulation and timing of this crucial step that appears more complicated than anticipated.

## Materials and Methods

### Cell lines

MEF cells were cultured at 37°C in the presence of 5% CO2 in Dulbecco's modified medium supplemented with 10% FCS.

### Quantitative UV survival analysis

Ten thousand cells were plated on a 3.5 cm Petri dish, cultured overnight and UV-irradiated with UV-C light (254 nm) at various doses (0.5 J/m^2^/s). After 4 days, cells were dried and stained by crystal violet, then lysed and quantified by spectrometry at 570 nm wavelength. When indicated, MEF cells were incubated with TSA (10 nM final concentration) for 3 hours before UV irradiation and during the 4 days of the post-irradiation period.

### Immunofluorescent-based DNA lesion quantification

Five thousand MEF cells were plated in 96 well plates (OptiPlates-96, Perkin Elmer). Twenty-four hours later, cells were UV-irradiated with UV-C lamp (10 J/m^2^) and recovered in fresh medium for the indicated period of time at 37°C, 5% CO2. Immuno-labeling of (6-4)PP was performed using mouse 64M-2 antibodies. DNA was denatured with 2 M HCl for 30 minutes at RT and blocked in 10% BSA in PBS for 15 minutes prior to labeling. (6-4)PP lesions were quantified using an IN Cell Analyser 1000 imaging system (GE Healthcare) and the percentage of (6-4)PP removal was determined (100% represents the amount of lesions determined just after UV irradiation).

### Unscheduled DNA synthesis (UDS) and transcription recovery after UV irradiation (RRS)

UDS was determined by counting the number of grains on at least 50 non-S-phase cells in autoradiographic preparations of cultures incubated for 3 h after UV-irradiation in medium containing 3H-thymidine (3H-TdR, Amersham, specific activity 25 Ci/mmol) [25]. In RRS, mock-treated or UV-irradiated cells (10 or 20 J/m^2^) were pulse labeled with 5 µCi/ml of [^3^H]uridine (PerkinElmer Life and Analytical Sciences, Boston MA 02118 USA) for 30 minutes, 24 h post-irradiation. Cells were fixed and auto-radiographied.

### Fluorescence recovery after photobleaching (FRAP)

Cell lines stably expressing GFP-RPB1^α-amaR^ were generated by transfection of MEF^WT^ or MEF^DOT1L^ with 1 ug of pAT7h1^α-amaR^ [36] using FuGENE6 (Roche Diagnostics, Mannheim, Germany). One day after transfection, cells expressing GFP-RPB1^α-amaR^ were selected by overnight incubation with 20 ug/ml of alpha-amanitin. Three days prior to microscopy experiments, cells were seeded onto 24 mm diameter coverslips. Imaging and FRAP were performed on a Zeiss LSM 710 meta confocal laser scanning microscope (Zeiss, Oberkochen, FRG).

FRAP analysis was performed at high time resolution as previously described [37]. Briefly, a strip spanning the nucleus was photo-bleached for 20 ms at 100% laser intensity (laser current set at 6.1 Å). We monitored the recovery of fluorescence in the strip every 20 msec for 20 sec at 0.5% of laser intensity. Twenty independent measurements were performed and the average values were used for every mobility curve. Mobility curves show relative fluorescence (fluorescence post-bleach divided by fluorescence pre-bleach) plotted against time. Error bars included in all the plotted FRAP data represent the SEM. Whenever two distinct FRAP curves were not easily dissociable, the statistical significance of their difference was checked by using Student's t-test (two-sample, two-tailed) within an appropriate time window: right after the photobleaching when evaluating mobility differences or after complete recovery when immobile fractions were being evaluated.

### Initiation-elongation transition assay *in vivo*

We grew cells overnight on 60 mm plates to 70–80% confluency and treated them with 100 µM of 5,6-Dichlorobenzimidazole 1-β-D-ribofuranoside (DRB) (Sigma) in culture medium for 3 hours. The cells were washed twice with PBS and incubated in fresh medium for various periods of time and RNA was extracted. Trichostatin A (TSA) (Sigma) was used at a concentration of 20 nM and was added 12 hours before treatment with DRB and maintained during the time of the experiment. When indicated, cells were UV-irradiated (15 J/m^2^) after the DRB treatment.

### Reverse transcription and real-time quantitative PCR

cDNA synthesis was performed by using hexamere and AMV reverse transcriptase (Sigma; St. Louis, MO). Real-time quantitative PCR was done with the FastStart DNA Master SYBR Green kit and the Lightcycler apparatus (Roche Diagnostic; Basel, Switzerland). Primer sequences are available upon request.

### ChIP

Cells were crosslinked with a 1% formaldehyde solution for 10 minutes at RT. Crosslinking was stopped by addition of glycine at 125 mM final concentration. Samples were sonicated to generate 500 bp DNA fragments. For immunoprecipitations, 100 µg of chromatin extract was pre-cleared for 2 hours with 50 µl of protein G-sepharose before addition of the indicated antibodies. Then, 2 µg of antibody was added to the reactions and incubated over night at 4°C in the presence of 50 µl of protein A/G beads. After serial washings, the immunocomplexes were eluted twice for 10 minutes at 65°C and crosslinking was reversed by adjusting to 200 mM NaCl and incubating 5 hours at 65°C. Further proteinase-K digestion was performed for 2 hours at 42°C. DNA was purified using Quiagen columns (QIAquick PCR Purification Kit). Immunoprecipitated DNA was quantified by real-time PCR. Primer sequences are available upon request.

### Host-cell reactivation assay

The pEGFP-reporter construct was purchased from Clontech. The pEGFP-reporter vector was UV-irradiated (254 nm, 600 J/m^2^) at a concentration of 1 µg/ml in 10 mM Tris-HCl (pH 8.0) and 1 mM EDTA. MEF cells were transfected with 3 µg of pEGFP-reporter and 1 µg of unirradiated pRED-reporter in a six-well plate at a confluence of 95% using X-tremeGENE 9 DNA Transfection Reagent (Roche). A 3/1 ratio was used to ensure that every cell expressing RFP protein expresses GFP in non-irradiated condition. After 12 hours of incubation, GFP and RFP were detected by reverse microscopy.

### Antibodies

Primary antibodies (the final dilutions are indicated in parentheses) used were anti-6–4PP (Cosmobio; 64M-2, dilution 1/500), anti-TFIIB C-18 (Santa Cruz, sc-225), anti-H3K79me1 (Abcam, ab2886) (1/1.000), anti-H3K79me3 (Abcam, ab2621) (1/1.000), anti Histone H3 (Abcam, ab1791), anti-GFP (Clinisciences, TP-401) and anti-DOT1L (Novus Biologicals, NB100-40845). Anti-Acetylated H4 is a mouse monoclonal antibodies produced at the IGBMC.

### Statistics

When required, the trapezoid rule was used to estimate the area under the curve (AUC). Student's t-test was used to assess whether the mean AUC (graph curves) or mean values from triplicate (bar graphs) were statistically significant. A P-value of 0.05 or less was considered as significant.

## Supporting Information

Data S1 Data of the Strip-FRAP curves of RNA Pol II-GFP protein stably expressed in either MEF^WT^ or MEF^DOT1L^ cells untreated (−UV) or treated (+UV) with UV (16 J/m^2^), 1 hour before photobleaching. Cells were photobleached with a 488 nm laser at maximum power 4 sec after the beginning of the acquisition. One image per 20 msec was taken during 40 sec in the photobleached area. Twenty independent measurements were performed and the average values were used for every mobility curve. SEM is indicated.
(XLSX)

Figure S1 Knocked-down of DOT1L induces UV sensitivity. (**A**) Ten µg of fractions from histone acid-extraction performed on MEF^WT^, MEF^DOT1L^, MEF^XPG^ or MEF^CSB^ cells were resolved by SDS-PAGE and Western-blotted for H3 or H3K79me3. (**B**) Total lysates were prepared from HeLa cells treated with control (siCTL), *XPA* (siXPA) or *DOT1L* (siDOT1L) siRNA. DOT1L, XPA and βTubulin were detected following SDS-PAGE and Western-blotting. siRNA smart pools are from Dharmacon. (**C**) HeLa cells treated with siCTL, siXPA or siDOT1L were irradiated with increasing doses of UV-C light. Cell survival was determined 96 h later, as detailed in the Experimental Procedures. Data were normalized to the mock treatment controls (as value of 1). The values are the means of three independent experiments +/− SD.
(PDF)

Figure S2 RNA pol II occupancy at housekeeping genes. (**A–B**) Time-dependent occupancy of RNA Pol II at the promoter of the *B2M (A) or KLF7 (B)* genes following UV irradiation (10 J/m^2^). Soluble chromatin was prepared from MEF^WT^ and MEF^DOT1L^ cells at indicated time points after UV-C treatment and subjected to ChIP assay. Real-time PCR using specific primers was performed to test the relative enrichment at the proximal promoters. The results are expressed as % of inputs. The values are the means of a triplicate experiment (± SD).
(PDF)
