# Supplementary figures and images for: Histone Methyltransferase DOT1L Drives Recovery of Gene Expression after a Genotoxic Attack
Source: PLoS Genet. 2013 Jul 4;9(7):e1003611. doi: 10.1371/journal.pgen.1003611 (PMC3701700; doi:10.1371/journal.pgen.1003611)

A

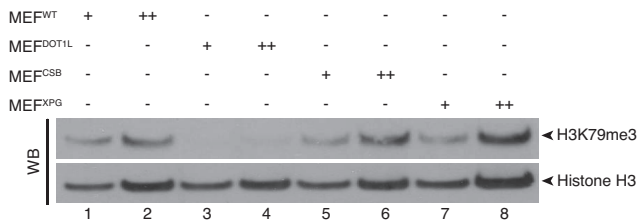

B

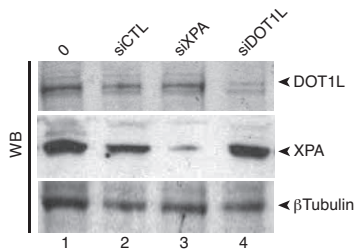

C

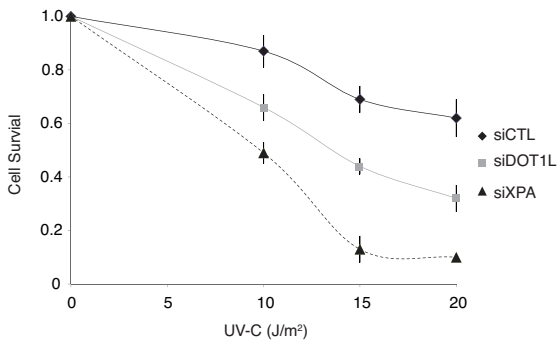

Supplement: Figure S1 — Knocked-down of DOT1L induces UV sensitivity. (A) Ten µg of fractions from histone acid-extraction performed on MEFWT, MEFDOT1L, MEFXPG or MEFCSB cells were resolved by SDS-PAGE and Western-blotted for H3 or H3K79me3. (B) Total lysates were prepared from HeLa cells treated with control (siCTL), XPA (siXPA) or DOT1L (siDOT1L) siRNA. DOT1L, XPA and βTubulin were detected following SDS-PAGE and Western-blotting. siRNA smart pools are from Dharmacon. (C) HeLa cells treated with siCTL, siXPA or siDOT1L were irradiated with increasing doses of UV-C light. Cell survival was determined 96 h later, as detailed in the Experimental Procedures. Data were normalized to the mock treatment controls (as value of 1). The values are the means of three independent experiments +/− SD. (PDF) [file pgen.1003611.s002.pdf]

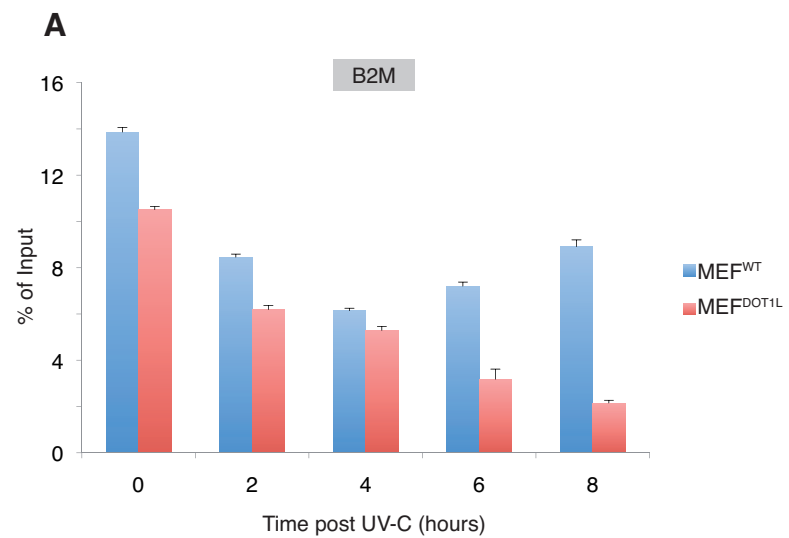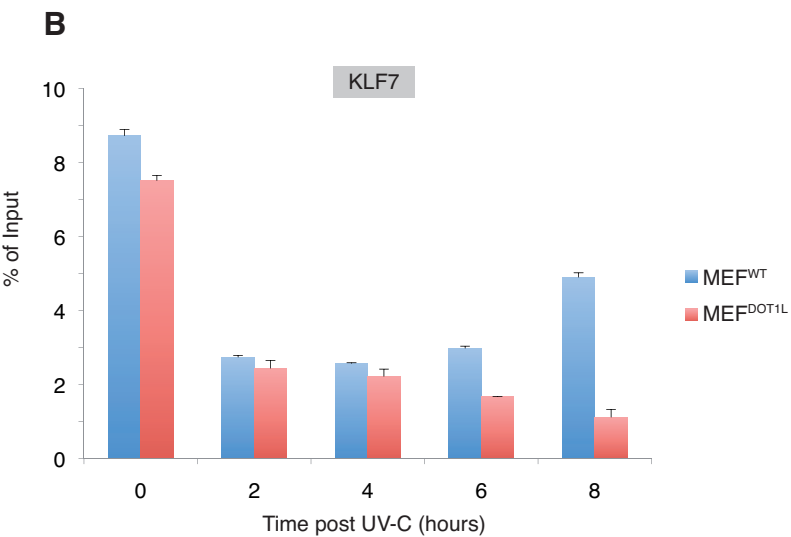

Supplement: Figure S2 — RNA pol II occupancy at housekeeping genes. (A–B) Time-dependent occupancy of RNA Pol II at the promoter of the B2M (A) or KLF7 (B) genes following UV irradiation (10 J/m2). Soluble chromatin was prepared from MEFWT and MEFDOT1L cells at indicated time points after UV-C treatment and subjected to ChIP assay. Real-time PCR using specific primers was performed to test the relative enrichment at the proximal promoters. The results are expressed as % of inputs. The values are the means of a triplicate experiment (± SD). (PDF) [file pgen.1003611.s003.pdf]
